# The design and evaluation of a shaped filter collection device to sample and store defined volume dried blood spots from finger pricks

**DOI:** 10.1186/s12936-015-0558-x

**Published:** 2015-02-05

**Authors:** Spencer D Polley, David Bell, James Oliver, Frank Tully, Mark D Perkins, Peter L Chiodini, Iveth J González

**Affiliations:** Department of Clinical Parasitology, Hospital for Tropical Diseases, Mortimer Market, Capper St, University College London NHS Foundation Trust, WC1E 6JB London, UK; Global Good, Intellectual Ventures Lab, 1555 132nd Ave NE, Bellevue, WA 98005 USA; 42 Technology Limited, Meadow Lane, St. Ives, Cambridgeshire PE27 4LG UK; FIND (Foundation for Innovative New Diagnostics), Campus Biotech, 9 Chemin des Mines, 1202 Geneva, Switzerland; London School of Hygiene & Tropical Medicine, Keppel Street, London, WC1E 7HT UK

**Keywords:** Dried blood spot, Malaria, Sampling, Shaped filter paper, DNA extraction, PCR, LAMP

## Abstract

**Background:**

Dried blood spots are a common medium for collecting patient blood prior to testing for malaria by molecular methods. A new shaped filter device for the quick and simple collection of a designated volume of patient blood has been designed and tested against conventional blood spots for accuracy and precision.

**Methods:**

Shaped filter devices were laser cut from Whatman GB003 paper to absorb a 20 μl blood volume. These devices were used to sample *Plasmodium falciparum* infected blood and the volume absorbed was measured volumetrically. Conventional blood spots were made by pipetting 20 μl of the same blood onto Whatman 3MM paper. DNA was extracted from both types of dried blood spot using Qiagen DNA blood mini or Chelex extraction for real-time PCR analysis, and PURE extraction for malaria LAMP testing.

**Results:**

The shaped filter devices collected a mean volume of 21.1 μl of blood, with a coefficient of variance of 8.1%. When used for DNA extraction by Chelex and Qiagen methodologies the mean number of international standard units of *P. falciparum* DNA recovered per μl of the eluate was 53.1 (95% CI: 49.4 to 56.7) and 32.7 (95% CI: 28.8 to 36.6), respectively for the shaped filter device, and 54.6 (95% CI: 52.1 to 57.1) and 12.0 (95% CI: 9.9 to 14.1), respectively for the 3MM blood spots. Qiagen extraction of 200 μl of whole infected blood yielded 853.6 international standard units of *P. falciparum* DNA per μl of eluate.

**Conclusions:**

A shaped filter device provides a simple way to quickly sample and store a defined volume of blood without the need for any additional measuring devices. Resultant dried blood spots may be employed for DNA extraction using a variety of technologies for nucleic acid amplification without the need for repeated cleaning of scissors or punches to prevent cross contamination of samples and results are comparable to traditional DBS.

**Electronic supplementary material:**

The online version of this article (doi:10.1186/s12936-015-0558-x) contains supplementary material, which is available to authorized users.

## Background

The wide scale usage of dried blood spots (DBS) was first introduced by Robert Guthrie in 1963 to facilitate neonatal screening for phenylketonuria [1]. These consisted of human blood applied directly onto an absorbent cotton fibre filter paper and air dried for several hours in order to immobilize the blood on the matrix and preserve it. The DBS were simple to produce and could easily be stored in sealed plastic bags and transported to the laboratory for subsequent processing, and even today are considered as non-infectious material by International Air Transport Association (IATA) regulations. Since the first introduction of the Guthrie test, the range of metabolic disorders, pharmacological studies, infectious diseases and basic biological investigations to which DBS have been applied has steadily increased [2-7] as have the methodologies used to process them. By contrast, the procedures used to generate many such DBS have remained practically identical to those first implemented by Guthrie.

For malaria, the usage of DBS for surveillance programmes remains particularly attractive. They provide the ability to collect, transport and store a large number of blood samples for subsequent analysis using serological [8] and nucleic acid based assays [9,10]. Traditionally a number of different matrices have been available for creation of malaria DBS, such as the cotton cellulose 3MM, 903, GB002, GB003, GB004 papers (Whatman, GB) and 226 sample collection devices (Perkin Elmer, USA), the glass fibre Wallac filter mat A (Perkin Elmer, USA) and GF/C (Whatman, GB) papers and the non cellulose BondElut Dried Matrix Spotting paper (Agilent Technologies, USA). Although, newer matrices such as FTA cards (Whatman, GB), HemaForm (available as a stand alone paper or within the HemaSpot device, Spot On Sciences, USA) and Mitra (Phenomenex, USA) have been developed to facilitate sample stabilization or recovery of biologically active molecules, a great many of the dried blood spots used in malaria programmes continue to employ cotton cellulose papers.

Blood may be applied to such filter papers by pressing the paper against a blood droplet at the site of a skin prick, in which case the volume of the blood may be crudely controlled by the diameter of DBS. Alternatively, the use of a defined diameter punch can also approximate the sampling of a set volume of blood from a given DBS, but several factors may impact on the precision of these techniques [11]. A set volume of blood can be measured volumetrically before application to the filter paper to ensure consistency of sample size, using small volume pipettes. In addition a variety of simple and cheap devices have been designed specifically for blood sampling to facilitate the use of rapid diagnostic tests (RDTs). These include marked straws, open cups and loops, however, a degree of inaccuracy has been reported when using such devices [12], and such devices may not measure the optimal volume for a given assay.

A number of nucleic acid extraction techniques have been developed for DBS, including methanol, Chelex, TE and enzymatic extraction [10,13], in addition to commercial products such as the Instagene (Biorad, UK,) ChargeSwitch Forensic DNA Purification (Invitrogen, USA) and DNA blood mini (Qiagen, Germany) kits [6]. Prior to the deployment of such protocols, the DBS is processed, either by cutting out some or all of the DBS with scissors or punching out one or more disks from it. In order to prevent cross contamination of samples the punch or scissors are extensively cleaned in ethanol and then used to make several cuts/punches in clean filter paper. Several automated extraction techniques have been published for the analysis of biological/pharmacological compounds, but all involve this manual step [14-16]. The cutting of blood spots has been automated by the use of a laser cutter [17], however, such a device is not yet readily available for widespread use and could conceivably be somewhat problematic to take into the field. Disposable punches eliminate the need for repeated cleaning of the punch, but consideration must be given to the financial cost of these devices and the disposal of the waste generated. Perforated DBS (pDBS) consist of a set volume of blood applied to the centre of a circle of perforations on a filter paper, which is then dried. Upon analysis the entire disc is pushed out of the filter paper using a disposable tip, eliminating the need for punches, but still requiring the use of a set volume sampling device in their generation [18]. The Mitra (RUO) microsampler (Phenomenex, USA) has been recently developed to sample and store a precise volume of blood, which can then be processed without the need of a punch. However, as yet the device is limited to a 10 μl volume and no data exists for its compatibility with DNA extraction.

Given the above limitations, a shaped filter device for blood sampling and transport has been developed that is cheap to manufacture and capable of quickly and easily sampling 20 μl of blood from a finger prick with good accuracy and precision. The device incorporates a stick of filter paper for handling the device, which is kept free of patient blood. This serves to minimize the potential for cross contamination during the manipulation samples (thereby eliminating the need for repeatedly cleaning the cutting device) whilst protecting the user from exposure to blood products. The device is compatible with Chelex, Qiagen and PURE DNA extraction methodologies.

## Methods

**Figure 1:**
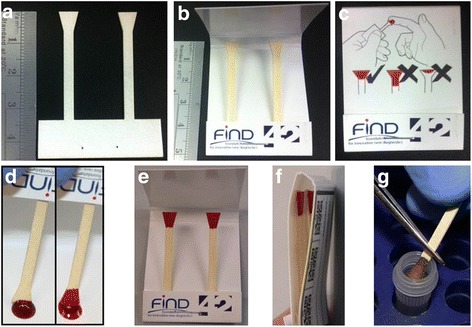
**Shaped filter device for sampling and archiving 20 μl** **of blood. a**, the shaped filter insert laser cut from GB003. **b**, the insert mounted inside a polyester coated, folded card sleeve. **c**, diagrammatic instructions for using the device printed on the sleeve. **d**, the device being filled by placing it against a blood spot until the inverted triangle shape is filled. **e** unfolded, filled devices left to air dry. **f**, folded, filled devices ready for storage. **g**, a single sample removed and cut for DNA extraction.

A commercial prototyping company was approached to laser cut a defined volume shape (Figure 1a) from Whatman GB003 paper, chosen for ease of cutting and manipulation, combined with its high wicking capacity. The shape comprised an inverted triangle of blotting paper (to absorb the patient’s blood) joined to a thin rectangle of blotting paper at its apex (for manipulation of the sample). A single device comprised two of these, assembled into a sleeve of polyester coated card (Figure 1b). The sleeve is pre-folded so as to minimize the pressure exerted by the card onto the sampling region of the device (Figure 1c and f), with visual instructions printed on the outside of the card to assist in its correct usage (Figure 1c). The devices were cut and then hand assembled in clean room conditions.

To assess the accuracy and precision of the prototype shaped filter blood collection device, a 50 μl spot of *Plasmodium falciparum* infected EDTA anticoagulated blood was placed onto a weigh boat on a laboratory balance and the balance zeroed. The blood was absorbed onto the collection device by touching the inverted triangle against the blood spot (Figure 1d). The volume of blood absorbed onto the device was controlled visually by the operator, who was blinded to the reading on the balance. When the blood reached the apex of the triangle (Figure 1d) the device was removed from the blood spot to prevent any more blood being absorbed. The volume of blood absorbed onto the collection device was determined by the weight of blood removed from the blood spot (1 mg = 1 μl). This volume was recorded and the droplet refreshed with 20 μl of blood. The balance was re-zeroed and the process repeated. After ten weightings the weigh boat was discarded and a fresh weigh boat used with a fresh 50 μl droplet. This process was repeated to generate a total of 80 separate measurements. In addition to these 80 samples, 80 traditional DBS were created by measuring 20 μl of the infected blood onto Whatman 3MM paper using a Gilson P20 pipette. The blood spots on the shaped filter device and 3MM paper were left to dry for four hours at room temperature (23 degrees C) in a class II microbiological cabinet with the fan on (Figure 1e). Following drying, they were stored in an air-tight box with silica gel desiccant at −20 degrees C prior to use. The blood used to create these DBS contained cultured 3D7 *P. falciparum* ring stage parasites. Parasites were synchronized in culture using sorbitol before being diluted down in uninfected O-negative blood to the required density, as determined by slide microscopy. Parasite density was checked by extraction of DNA from two 200 μl aliquots of the resultant blood and real-time PCR analysis of 2 μl of the DNA eluate as detailed below. The eluates were found to contain 853.6 international standard units of parasites DNA per μl using a real-time PCR targeting chromosomal loci [19].

52 DBS on shaped filter devices were used for DNA extraction; 28 were processed using a Chelex DNA extraction protocol [10], and 24 were extracted with a DNA blood mini kit (Qiagen, Germany) as per the manufacturer’s protocols for DBS. The same number of traditional DBS on 3MM paper was also extracted at the same time using these same methodologies. Purified DNA was used to quantify malaria DNA content using a real-time PCR assay targeting the chromosomal 18S locus [19] and a second in-house assay targeting the mitochondrial DNA [20] that was converted into a real time format by the use of 1x QuantiTect SYBR green master mix [see Additional file 1]. Assays were run on a Rotorgene-Q (Qiagen, Germany). DNA was quantified against Qiagen DNA mini-prep extracted samples of the 1^st^ WHO International Standard for *P. falciparum* [21] diluted into uninfected human blood. The number of IUs per μl of extract from these standards was calculated assuming a 100% recovery of DNA into the eluate. Samples were run in duplicate and the reference standard curve and parasite concentration for DNA extracted from DBS calculated automatically by the Rotor-Gene Q Series Software, version 2.0.2 (Build 4) (Qiagen, Germany). The mean parasite DNA concentration (in international IUs), standard deviation and coefficient of variance were calculated for each set of replicate blood spots processed with either Chelex or Qiagen extraction methods.

DNA from 24 shaped filter DBS, 24 traditional 3MM DBS and 24 20 μl blood samples was also extracted using the PURE DNA clean up technology prior to their analysis using the commercially available malaria LAMP kit (Loopamp™ MALARIA Pan/Pf detection kit - LMC 562, Eiken Chemical Co., Ltd. Tokyo, Japan) as per published standard operating procedures [22]. LAMP was performed in an LA320CE real-time turbidimeter. The mean time to positive turbidity (Tt) for each sample was converted into DNA concentration (expressed as IUs per μl) using a standard curve of Tts generated from PURE extracted DNA isolated from dilutions of the international *P. falciparum* standard in uninfected human blood. These dilutions were created to contain 10,000, 2,000, 400, 80 or 16 IU per μl blood and the resulting PURE extracts were run in triplicate using the Pf LAMP malaria test with a logarithmic standard curve fitted to the output using Excel. The 200 μl of eluate was assumed to contain 100% of the DNA in the original 20 μl of blood for compilation of the standard curve. Converted Tts were used to calculate the mean concentration of DNA per μl of extract for each type of sample based, together with its standard deviation and coefficient of variation. The amount of DNA extracted from each type of blood spot and whole blood were compared using unpaired t-tests assuming equal variance.

## Results and discussion

80 separate DBS generated using the shaped filter device absorbed a mean blood volume of 21.1 μl when analysed volumetrically, with a standard deviation of 1.7 μl. This equates to a coefficient of variance of 8.1%. Based upon these data 90%, 95% and 99% of fills are expected to lie within +/−2.8 μl, +/−3.4 μl and +/−4.4 μl of the mean, respectively.

When used for Chelex DNA extraction, DBS created using the shaped filter device yielded a mean concentration of 53.1 (95% CI: 49.4 to 56.7) ISUs of parasite chromosomal DNA per μl of eluate with a CV of 17.7% (Table 1). In 28 replicate extractions the minimum and maximum parasite concentrations per μl of eluate were 33.5 and 72.5 ISUs of parasite chromosomal DNA. The traditional blood spots on 3MM paper gave an almost identical yield of parasite chromosomal DNA, with a mean concentration of 54.6 (95% CI: 52.1 to 57.1) ISUs per μl of eluate. The CV of these eluates was 12.0% and in the 28 replicate extractions the minimum and maximum concentrations of parasite chromosomal DNA were 40.8 and 65.2 ISUs per μl of eluate. A *t*-test for unpaired samples with equal variances showed no significant difference in the level of chromosomal DNA extracted from the two types of DBS (P = 0.461).

**Table 1:** **DNA concentration per** μ**l of eluate determined in International Standard Units from Chelex and Qiagen extracted blood spots and whole blood**

	**Mean ISU per** **μl eluate**	**95% CI**	**Coefficient of variation**	**Minimum ISU per** **μl eluate**	**Maximum ISU per** **μl eluate**	***t*** **-test result**
Chromosomal target (18 s)						
Chelex GB003	53.1	(49.4 to 56.7)	17.7%	33.5	72.5	
Chelex 3MM	54.6	(52.1 to 57.1)	12.0%	40.8	65.2	0.461
Qiagen GB003	32.7	(28.8 to 36.6)	30.5%	11.5	49.6	
Qiagen 3MM	12.0	(9.9 to 14.1)	45.2%	3.2	27.5	<0.001
200 μ Raw blood	853.6	(790.3 to 917.0)				
Mitchondrial target						
Chelex GB003	62.7	(56.5 to 68.9)	24.9%	41.5	105.5	
Chelex 3MM	84.9	(80.2 to 89.6)	14.0%	58.3	103.2	<0.001
Qiagen GB003	63.3	(59.8 to 66.9)	13.3%	44.2	75.6	
Qiagen 3MM	42.7	(38.6 to 46.8)	22.5%	23.7	60.5	<0.001
200 μl Raw blood	1718.8	(1640.8 to 1796.8)				

Two separate real-time PCRs were used to assay chromosomal (18 s) and mitochondrial loci. The P values were generated by comparing the level of DNA eluted from 3MM and GB003 with Chelex or Qiagen DNA mini extractions using an unpaired *t*-test. Assumptions of equal variance were tested by ensuring there was less than a twofold difference between the standard deviations of the two groups compared.

By comparison, when Qiagen blood DNA mini columns were used to extract DNA, significantly more chromosomal DNA was eluted from the shaped filter compared to the traditional 3MM papers. The respective mean DNA concentrations were 32.7 (95% CI: 28.8 to 36.6) and 12.0 (95% CI: 9.9 to 14.1) ISUs of parasite chromosomal DNA per μl of eluate. The CV of the eluates extracted from the shaped filter device and traditional 3MM paper were 30.5% and 45.2% respectively and the minimum and maximum yields of parasite chromosomal DNA were 11.5 and 49.6, and 3.2 and 27.5 ISUs respectively. A *t*-test for unpaired samples with unequal variances showed the shaped filter paper yielded significantly more malarial chromosome DNA than blood spots on 3MM paper (P < 0.0001).

DNA eluted from the two types of DBS was also compared using a real-time assay targeted to mitochondrial DNA (Table 1). In this assay, shaped filter papers again produced significantly more DNA than the traditional DBS when Qiagen technology was used in the extractions (P < 0.0001). For Chelex extractions, however, traditional DBS gave a significantly higher yield of parasite mitochondrial DNA compared to shaped filter papers (P < 0.0001). It is worth noting, however, that mean DNA yield from the shaped filter paper was only 28% lower than that from the traditional DBS. Further optimization of the extraction protocol for shaped filter papers may be possible for mitochondrial targets.

Of significant interest for malaria screening programmes is the relative amounts of DNA recovered from DBS using the Chelex procedure compared to whole blood subject to Qiagen extraction. The concentration of chromosomal DNA released by Chelex extraction of a 20 μl blood spot was less than 7% of that produced from the 200 μl whole blood sample (853.6 ISUs of *P. falciparum* DNA per μl of eluate). This is not surprising in light of disparity of blood volumes processed by each methodology; given both methods produce approximately the same volume of DNA eluate, the maximum theoretical DNA concentration that may be liberated from the Chelex processed DBS is tenfold less than could be achieved using whole blood. Similar reductions in DNA recovery between blood and filter papers have been reported for human DNA [22], whilst DBS have been shown to be qualitatively less good than whole blood for malaria diagnosis [23]. This reduction in DNA concentration may impose a significant limitation to the level of sensitivity that can be achieved using DBS in epidemiological screening or “Find and Treat” programmes unless more sensitive targets can be utilized for amplification [24]. Where DBS and PCR have to be used, then Chelex extraction would seem to outperform Qiagen based DBS processing for detection of malarial DNA, a phenomenon observed by other groups [9].

In the LAMP analysis, the mean concentration of *P. falciparum* DNA (in IUs per μl of PURE extract) was 102.5 (95% CI: 89.5 to 115.5) for whole blood, 36.2 (95% CI: 33.7 to 38.6) for 3MM DBS and 20.7 (95% CI: 17.1 to 24.2) for the shaped filter papers (Table 2). PURE eluates derived from both sets of filter papers contained a significantly lower number of IUs than PURE eluates derived from whole blood (P < 0.001 for both sets of eluates). In real terms 3MM and GB003 based DBS yielded 36% and 20% of the level derived from whole blood.

**Table 2:** **DNA concentration per** μ**l of eluate determined in International Standard Units from PURE extracted DBS and whole blood**

	**Mean IU per** **μl eluate**	**95% CI**	**Coefficient of variation**	**Min IU per** **μl eluate**	**Max IU per** **μl eluate**	***t*** **-test result**
GB003	20.7	(17.1 to 24.2)	17.50%	7.7	35.5	<0.001
3MM	36.2	(33.7 to 38.6)	12.10%	26.3	46.3	<0.001
Raw blood	102.5	(89.5 to 115.5)		57.4	152.8	

The Time to positive turbidity (Tt) achieved for each extract was converted into International Units (IUs) of DNA per μl of eluate by comparison to a standard curve generated from PURE extracted blood containing different concentrations of the international standard for *P. falciparum*. The P values were generated by comparing the level of DNA extracted from 3MM DBS and shaped filter devices (P < 0.001) or 3MM DBS and whole blood (P < 0.001) using two separate unpaired t-tests. Assumptions of equal variance were tested by ensuring there was less than a twofold difference between the standard deviations of the two groups compared before the tests were performed.

The malaria LAMP kit has been shown to have an LOD of 0.5 parasites per μl of whole blood when using whole infected blood in the PURE extraction process [23,24] a figure comparable to that achieved with nested PCR and Qiagen purified DNA isolated from whole blood [25,26]. The best level of DNA extraction achieved with PURE extraction and DBS was 33% of the level achieved using whole blood, which should equate to an LOD of 1.5 parasites per μl of patient’s blood. By comparison, with only a 7% efficiency of DNA recovery from DBS using Chelex extraction (compared to Qiagen processing of whole blood), the theoretical LOD achievable with DBS and nested PCR would be just over 7 parasites per μl of patient blood. It would seem, therefore, that where it is necessary to collect samples as DBS for malaria surveillance or elimination, LAMP would appear to be superior to PCR in the detection of low level infections (although this remains to be empirically tested). In addition the PURE system can utilize wet as well as dried blood spots. The PURE system has been developed to allow the rapid (<20 minute) clean up of DNA for LAMP in resource poor settings, but LAMP may be used with other DNA sources including Chelex and Qiagen extracted DNA where time constraints allow. When 5 μl of DNA extracted from GB003 paper by the Chelex method was used in the LAMP reaction the resultant Tts were comparable to those obtained with PURE DNA from whole blood [See Additional file 2]. It is feasible that an LOD of 0.5 parasites per μl would be achievable with this clean up system if time permitted the drying of filter papers and their overnight processing in saponin (these steps being integral to Chelex clean up). 12.5 μl of Qiagen DNA showed an equivalent output to that obtained with PURE extracted GB003 papers.

It should be noted that these data were generated with a stringent protocol for the creation of traditional DBS, namely the application of a defined volume of blood to the filter paper measured using a calibrated low volume pipette. The use of other devices such as straws or attempting to control the volume by regulating the diameter of the blood spot will in all likelihood significantly increase the variance of the blood volume sampled using traditional DBS. The use of a defined diameter punch will only partially mitigate this since blood is not evenly distributed across the filter paper in DBS [27,28], and protocols which sample a defined fraction of the blood spot (such as half or quarter of the DBS) can in no way control for variability in blood spot size and, therefore, the volume of blood assayed.

One significant difference between the shaped filter paper and the traditional 3MM filter paper is the time taken to process the sample for DNA extraction. The elimination of ethanol flaming, cleaning scissors and manually cutting blood spots reduces the processing time of each DBS from 45 to 15 seconds, whilst cross contamination is minimized by cutting a region of the device not contaminated with patient blood. This paper stick makes the sample much easier to handle, can be torn away from a sleeved device (Figure 1g) and protects the user from contamination with any blood products. The prototype device had diagrammatic instructions printed on the outside of the sleeve to aid its correct use (Figure 1c). The rear of the device had a section for filling in patient details together with Quick Response Code stickers (Figure 1f) to allow electronic information on a given patient to be linked to the relevant sample device. Such information could conceivably be entered using a smart phone app during sample collection, and include information such as a photograph to ensure that any treatments triggered by a positive result are targeted to the correct patient.

With a limited run of 5,000, it is estimated that the combined cost for materials and manufacture of each sampling device would be under 0.05 GBP (comprising the device alone with no sleeve). Sleeved versions of the sampling device would cost 0.30 GBP (hand assembled with a polyester coated, pre-folded card sleeve). These prices could well be reduced by manufacturing in bulk. Alternatively, the ability to manufacture the insert alone may represent a cost effective way for users to produce their own sleeved devices on site. The flexibility of laser cutting means that the device could be easily and cheaply adapted to allow different blood volumes or additional replicate samples to be collected according to a user’s particular requirements.

## Conclusions

Under these set of controlled conditions, it can be seen that the shaped filter paper provides a quick and simple way to sample and store blood in the form of dried blood spots. The device performed well with Chelex, Qiagen and PURE DNA extraction protocols compared to traditional DBS and provides a level of precision and accuracy comparable to a set volume of blood applied to 3MM paper using small volume pipettes. The next steps will be to test the device with blood from finger pricks in a malaria endemic location to evaluate its utility for field based serological and nucleic acid based sampling.

## Additional files

Additional file 1:
**Real-time PCR for detection of Plasmodium mitochondrial DNA.** The data describe the conditions used for the mitochondrial based real-time PCR reaction. Additional file 2:
**Time to positive turbidity (Tt) and International units per μl of LAMP reaction as determined by use of the WHO international standard for** ***P. falciparum.*** The data present the time to positive turbidity (Tt) obtained in LAMP reaction utilizing the DNA prepared by PURE, Qiagen and Chelex methodologies. The Tts were converted into International Units (IUs) of malaria DNA utilizing a standard curve generated from the WHO international standard for *P. falciparum*, and were used to calculate the amount of parasite DNA in IUs present in each μl of the LAMP reaction.

## Abbreviations

DBS: Dried blood spot
ISUs: International standard units
μl: Microlitre
