# Supplementary material for: The design and evaluation of a shaped filter collection device to sample and store defined volume dried blood spots from finger pricks
Source: Malar J. 2015 Feb 5;14:45. doi: 10.1186/s12936-015-0558-x (PMC4327959; doi:10.1186/s12936-015-0558-x)
Supplement: Additional file 1: — Real-time PCR for detection of Plasmodium mitochondrial DNA. The data describe the conditions used for the mitochondrial based real-time PCR reaction. [file 12936_2015_558_MOESM1_ESM.docx]

| Constituent | Sequence/Notes | Final concentration/ volume (in 20 mcl reaction) |
| --- | --- | --- |
| PgMtID19F3 | TCGCTTCTAACGGTGAAC | 1µM |
| PgMtID19B3 | AATTGATAGTATCAGCTATCCATAG | 1µM |
| 2x QuantiTect SYBR Green PCR Master Mix | Qiagen, de Hilden. | 10 mcl |
| DNA |  | 2 mcl |

**Real time PCR targeting the mitochondrial DNA of Plasmodium species**. A 20 mcl PCR reaction was set up as shown and then subjected to an initial hot start reaction of 95 ^o^C for 15 minutes. This was followed by 45 cycles of amplification: 95 ^o^C for 15 seconds, 55 ^o^C for 30 seconds and 68 ^o^C for 30 seconds, collecting data on the SYBR green channel.
