# Supplementary material for: The design and evaluation of a shaped filter collection device to sample and store defined volume dried blood spots from finger pricks
Source: Malar J. 2015 Feb 5;14:45. doi: 10.1186/s12936-015-0558-x (PMC4327959; doi:10.1186/s12936-015-0558-x)
Supplement: Additional file 2: — Time to positive turbidity (Tt) and International units per μl of LAMP reaction as determined by use of the WHO international standard for P. falciparum. The data present the time to positive turbidity (Tt) obtained in LAMP reaction utilizing the DNA prepared by PURE, Qiagen and Chelex methodologies. The Tts were converted into International Units (IUs) of malaria DNA utilizing a standard curve generated from the WHO international standard for P. falciparum, and were used to calculate the amount of parasite DNA in IUs present in each μl of the LAMP reaction. [file 12936_2015_558_MOESM2_ESM.docx]

| Template | Time to positive turbidity (Tt) | International Units of malaria DNA per μl of reaction reaction |
| --- | --- | --- |
| PURE extracted whole blood | 13.72 (13.80 – 13.64) | 102.5 (89.5 – 115.5) |
| PURE extracted GB003 | 14.71 (15.14 – 14.28) | 26.1 (6.3 – 45.9) |
| Chelex extracted GB003 | 13.73 (14.07 – 13.38) | 119.2 (54.6 – 183.8) |
| Qiagen extracted GB003 | 14.78 (15.10 - 14.46) | 20.4 (7.0 – 33.8) |

Time to positive turbidity (Tt) and International units per μl of LAMP reaction as determined by use of the WHO international standard for *P. falciparum*. 8 replicates of Chelex, Qiagen and PURE DNA extracted from GB003 blood spots were used to generate this data on a single run. The blood spots held 20 μl of whole blood containing 465 parasites per μl. 5 μl of Chelex or 12.5 μl of Qiagen extracted DNA was used in each 30 μl LAMP reaction. These reactions were supplemented by the use of 20 mM NaCl (final concentration). 30 μl of PURE extract was used per reaction for eluates from both blood spots and the whole blood. For PURE extracted whole blood, 20 μl of whole blood containing 465 parasites per μl was added to the PURE device, as shown in Table 2.
